# A novel mineralocorticoid receptor antagonist, 7,3',4'-trihydroxyisoflavone improves skin barrier function impaired by endogenous or exogenous glucocorticoids

**DOI:** 10.1038/s41598-021-91450-6

**Published:** 2021-06-07

**Authors:** Hanil Lee, Eun-Jeong Choi, Eun Jung Kim, Eui Dong Son, Hyoung-June Kim, Won-Seok Park, Young-Gyu Kang, Kyong-Oh Shin, Kyungho Park, Jin-Chul Kim, Su-Nam Kim, Eung Ho Choi

**Affiliations:** 1 Department of Dermatology, Yonsei University Wonju College of Medicine, 20 Ilsan-ro, Wonju, 26426 Republic of Korea; 2 Research and Development Center, AMOREPACIFIC, Yongin, Republic of Korea; 3 Department of Food Science and Nutrition, Convergence Program of Material Science for Medicine and Pharmaceutics, Hallym University, Chuncheon, Republic of Korea; 4 Natural Product Informatics Research Center, Korea Institute of Science and Technology, Gangneung, Republic of Korea

**Keywords:** Skin manifestations, Adverse effects

## Abstract

Excess glucocorticoids (GCs) with either endogenous or exogenous origins deteriorate skin barrier function. GCs bind to mineralocorticoid and GC receptors (MRs and GRs) in normal human epidermal keratinocytes (NHEKs). Inappropriate MR activation by GCs mediates various GC-induced cutaneous adverse events. We examined whether MR antagonists can ameliorate GC-mediated skin barrier dysfunction in NHEKs, reconstructed human epidermis (RHE), and subjects under psychological stress (PS). In a preliminary clinical investigation, topical MR antagonists improved skin barrier function in topical GC-treated subjects. In NHEKs, cortisol induced nuclear translocation of GR and MR, and GR and MR antagonists inhibited cortisol-induced reductions of keratinocyte differentiation. We identified 7,3’,4’-trihydroxyisoflavone (7,3’,4’-THIF) as a novel compound that inhibits MR transcriptional activity by screening 30 cosmetic compounds. 7,3’,4’-THIF ameliorated the cortisol effect which decreases keratinocyte differentiation in NHEKs and RHE. In a clinical study on PS subjects, 7,3',4'-THIF (0.1%)-containing cream improved skin barrier function, including skin surface pH, barrier recovery rate, and stratum corneum lipids. In conclusion, skin barrier dysfunction owing to excess GC is mediated by MR and GR; thus, it could be prevented by treatment with MR antagonists. Therefore, topical MR antagonists are a promising therapeutic option for skin barrier dysfunction after topical GC treatment or PS.

## Introduction

Psychological stress (PS) negatively affects epidermal barrier function^1,2^ and aggravates many cutaneous dermatoses, such as atopic dermatitis (AD) and psoriasis^3–8^. PS inhibits the proliferation and differentiation of keratinocytes and decreases the production and secretion of lamellar bodies and the density of corneodesmosomes, compromising both permeability barrier homeostasis and stratum corneum (SC) integrity^1,9^. PS-induced alterations in epidermal barrier homeostasis are mediated by increased cortisol, the endogenous glucocorticoids (GCs)^10^. The source of cortisol under PS is not only the activation of the hypothalamus–pituitary–adrenal (HPA) axis^11,12^ but also elevated 11β-hydroxysteroid dehydrogenase type I (11β-HSD1) in the skin^13^, which converts inactive cortisone into cortisol^14^. Likewise, systemic or topical administration of exogenous GC disrupts the skin barrier through a similar mechanism^15^.

The mineralocorticoid receptor (MR) and GC receptor (GR) are members of the same nuclear receptor superfamily^16^. Owing to their high structural similarity, not only the mineralocorticoid hormone, aldosterone, but also cortisol, can bind to MR with high affinity^17,18^. The binding of cortisol to MR is controlled at the pre-receptor level by 11β-hydroxysteroid dehydrogenase type II (11β-HSD2), which converts cortisol into inactive cortisone^19^. However, the epidermis is a well-recognized 11β-HSD2-deficient tissue^20,21^; thus, MR can be inappropriately activated by increased GCs^19,22–24^. Recent studies have highlighted that inappropriately activated MR caused by excess GC is involved in generating GC-mediated cutaneous side effects, such as delayed wound healing, epidermal atrophy, and skin aging. Furthermore, such side effects can be prevented by topical MR blockade^25–28^.

Therefore, we hypothesized that MR inappropriately activated by GC mediates skin barrier dysfunction caused by exogenous GC or PS, and thus, topical MR antagonism could prevent their detrimental effects on the skin barrier.

First, we conducted a brief preliminary clinical investigation to verify whether topical MR antagonists could prevent topical GC-induced skin barrier dysfunction. Second, we examined whether cortisol activates both GR and MR, and whether the receptors are inhibited by their own antagonists in normal human epidermal keratinocytes (NHEKs). To gain mechanistic insights into how MR antagonists improve the skin permeability barrier, the mRNA expression of epidermal differentiation markers was analyzed in NHEKs and immunohistochemical staining for epidermal differentiation markers was conducted in reconstructed human epidermis (RHE) after treatment with either cortisol or an antagonist of both GR and MR. Third, to find a novel compound that can be utilized as a cosmetic ingredient, we screened 30 compounds, selected one, and validated it as a novel MR antagonist. Finally, we conducted a clinical investigation to verify the effect of our newly developed MR antagonist in improving skin barrier function in PS participants.

## Results

### Topical application of spironolactone, an MR antagonist prevents topical GC-induced skin barrier impairment

**Figure 1:**
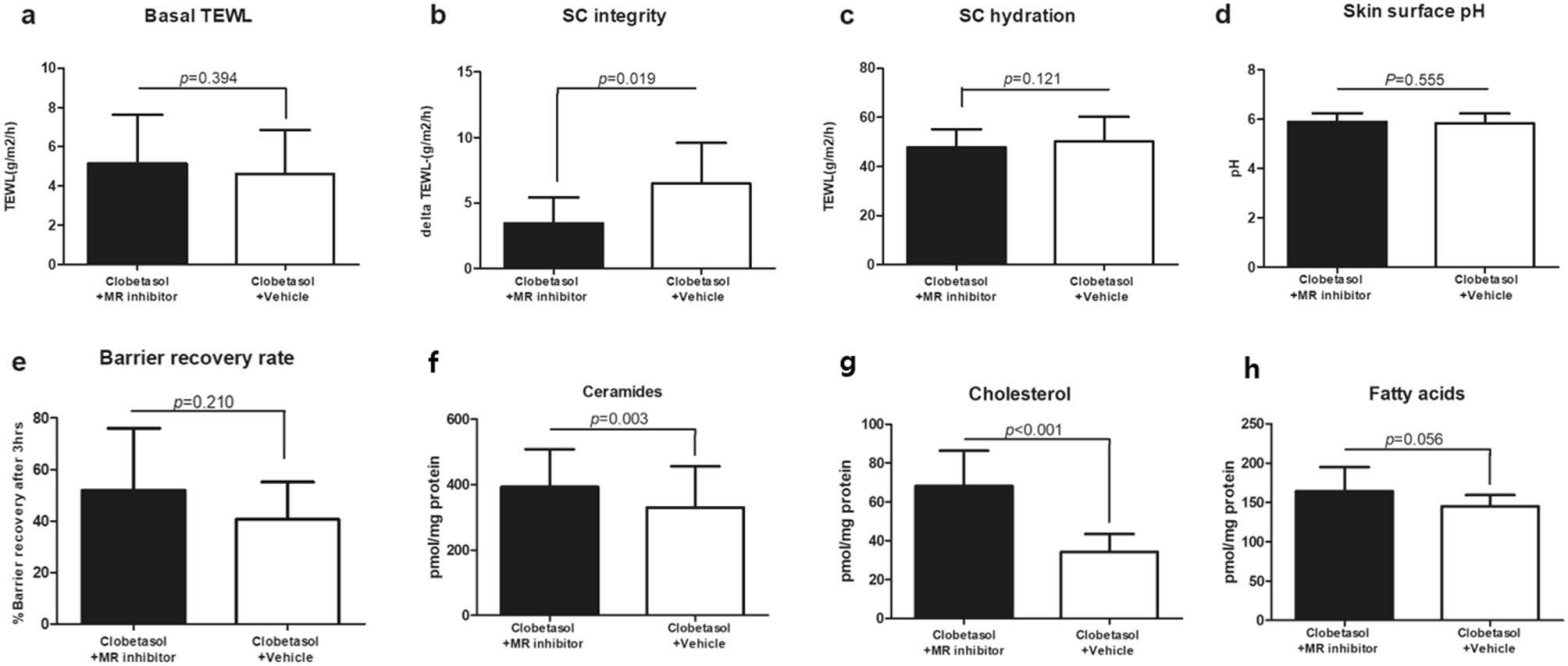
Topical application of spironolactone, a mineralocorticoid receptor antagonist, improves skin barrier function in clobetasol-treated skin and increases the amount of stratum corneum (SC) lipids. Comparison of basal transepidermal water loss (TEWL) (**a**), SC integrity (**b**), SC hydration (**c**), skin surface pH (**d**), and barrier recovery rate (**e**) between clobetasol + spironolactone-treated sides and clobetasol + vehicle-treated sides in healthy participants. Quantitative analysis of SC lipids, ceramides (**f**), cholesterol (**g**), and fatty acids (**h**) using tape-stripped skin samples from the participants. Data are expressed as mean ± SD (N = 11). Wilcoxon signed-rank test was used.

To preliminarily investigate the effect of an MR antagonist in improving skin barrier function that is impaired by GC, a small-sample clinical experiment was conducted using topical spironolactone, a proven MR antagonist, and topical GC. In 11 healthy participants, 0.05% clobetasol propionate ointment (Dermovate ointment; GlaxoSmithKline, Uxbridge, UK) was applied to both sides of the forearms, with 5% topical spironolactone cream (S5 cream, Shanghai Sunshine Technology, Shanghai, China) or Cetaphil moisturizing lotion (Galderma Laboratories, Fort Worth, TX, USA) as a control vehicle, on each side. After 5 days of topical application, the skin barrier function of the clobetasol + spironolactone-treated and clobetasol + vehicle-treated sides were measured and compared (Fig. 1a–e). Basal transepidermal water loss (TEWL), SC hydration, skin surface pH, and barrier recovery rate were not significantly different between the two sides. However, co-application of topical spironolactone resulted in significantly lower delta TEWL (*p* = 0.019). SC integrity was measured as the delta TEWL before and after tape-stripping 15 times on the same site on the skin. Each tape stripping removed the outer layer of the SC. Thus, the lower the value, the firmer the structure of the SC. In other words, SC integrity was improved by co-application with topical spironolactone.

To investigate the effect of MR antagonists on permeability barrier homeostasis, SC lipids were quantified using tape-stripped skin samples. Co-application of topical spironolactone significantly increased the amounts of cholesterol (*p* < 0.001) and to a much lesser extent of ceramides (*p* = 0.003) and fatty acids (FAs; *p* = 0.056), albeit without statistical significance (Fig. 1f–h). In summary, topical application of spironolactone, a proven MR antagonist, improved skin barrier function and increased the amounts of ceramides, cholesterol, and fatty acids in the SC of topical GC-treated skin.

### GC activates nuclear translocation of GR and MR in NHEKs

**Figure 2:**
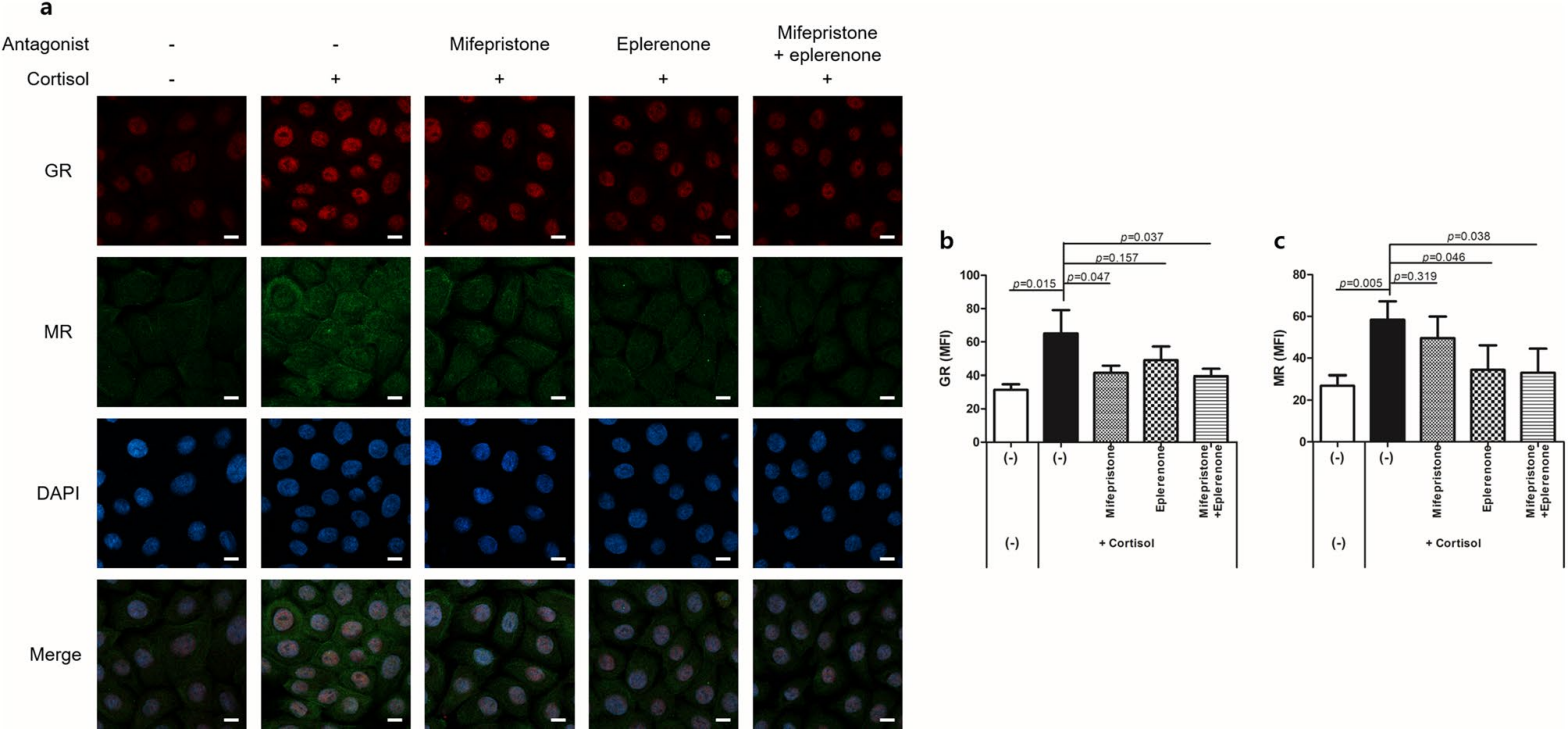
Cortisol activates nuclear translocation of glucocorticoid receptor (GR) and mineralocorticoid receptor (MR) in NHEKs. NHEKs were treated with mifepristone (GR antagonist, 1 μM) or eplerenone (MR antagonist, 1 μM) in the presence of cortisol (10 μM) for 24 h. Cells were stained with antibodies against GR (red) and MR (green) and observed under confocal microscopy. Nuclei were stained with DAPI (blue). (**a**) Representative images. Scale bars, 10 μm. (**b**, **c**) Quantification of mean fluorescence intensity (MFI) of GR and MR in nucleus. Data are expressed as mean ± SD of three independent experiments.

To determine whether cortisol activates both GR and MR, and whether the receptors are inhibited by their own antagonists, we examined the nuclear translocation of GR and MR in NHEKs treated with mifepristone (a GR antagonist) or eplerenone (an MR antagonist) in the presence or absence of cortisol (Fig. 2 and S1). Cortisol induced the nuclear translocation of both GR and MR. Mifepristone decreased the cortisol-induced nuclear signals of GR and eplerenone decreased the cortisol-induced nuclear signals of MR. Treatment with both mifepristone and eplerenone decreased the nuclear signals of both receptors. In the basal state, mifepristone slightly decreased the nuclear signals of GR, albeit without statistical significance (Fig. S1). However, eplerenone did not decrease those of MR. Co-treatment with mifepristone and eplerenone decreased the nuclear signals of GR, even in the basal state. These results indicate that basal level nuclear translocation of GR is present even without exogenous cortisol treatment. In contrast, basal level nuclear translocation of MR is relatively minimal compared to that of GR.

### GR and MR antagonists improve keratinocyte differentiation under GC treatment

**Figure 3:**
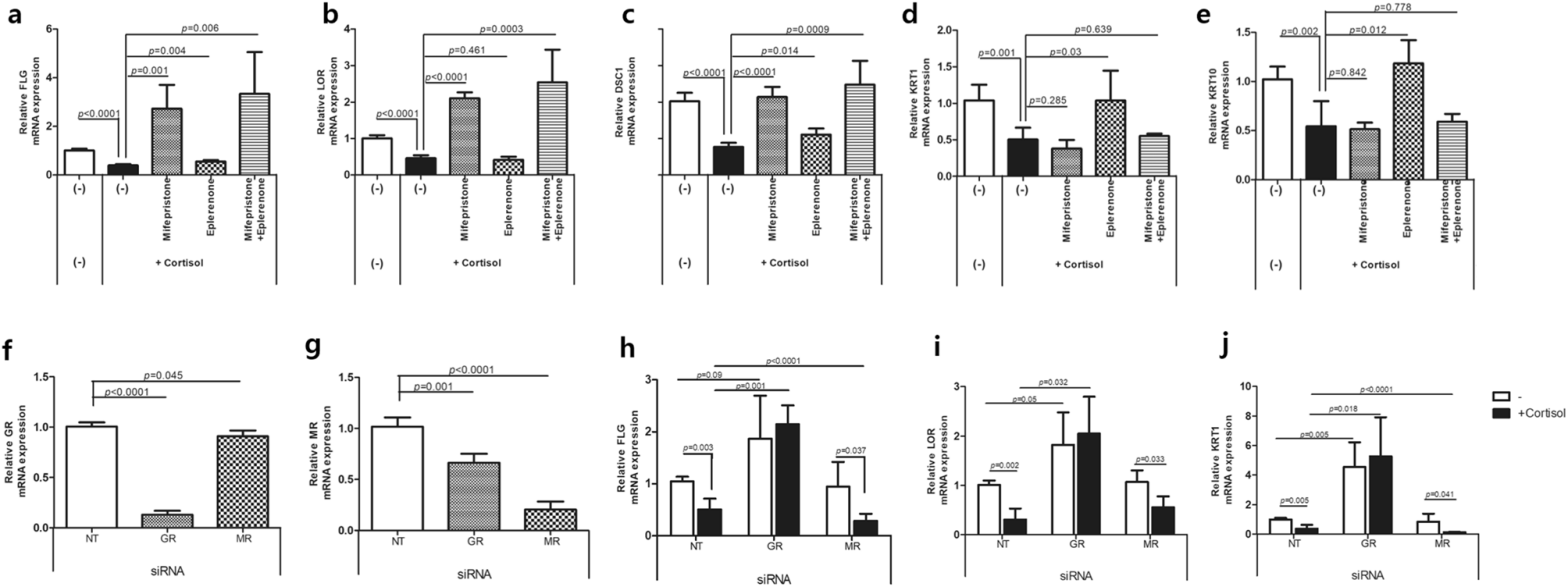
Effect of cortisol, GR antagonist, and MR antagonist on mRNA expression of keratinocyte differentiation markers in NHEKs. (**a**–**e**) NHEKs were treated with mifepristone (GR antagonist, 1 μM) or eplerenone (MR antagonist, 1 μM) in the presence of cortisol (10 μM) for 4 days, and the mRNA expression of differentiation markers, FLG, LOR, DSC1, KRT1, and KRT10 was analyzed by RT-qPCR. (**f–j**) NHEKs were transfected with control (non-targeting; NT), GR, or MR siRNAs. (**f**, **g**) mRNA level of GR and MR in siRNA-treated NHEKs. (**h**–**j**) mRNA level of FLG, LOR, and KRT1 in siRNA-treated NHEKs treated with cortisol (10 μM) for 4 days. mRNA levels were normalized to that of RPL13A. Data are expressed as mean ± SD of three independent experiments.

To examine the effect of cortisol on keratinocyte differentiation, and whether it is inhibited by antagonists of GR and MR, the mRNA levels of keratinocyte differentiation markers were analyzed in NHEKs treated with mifepristone and eplerenone in the presence or absence of cortisol. Cortisol reduced the mRNA levels of Filaggrin (*FLG*), Loricrin (*LOR*), Desmocollin-1 (*DSC1*), Keratin 1 (*KRT1*), *KRT10* and (Fig. 3a–e). Compared to the cortisol-only treatment group, co-treatment with mifepristone increased the mRNA levels of *FLG*, *LOR*, and *DSC1*, whereas co-treatment with eplerenone resulted in a remarkable increase in *KRT1* and *KRT10*, and a slight increase in *FLG* and *DSC1*. Co-treatment with both mifepristone and eplerenone potentiated the increases in the mRNA levels of *FLG*, *LOR*, and *DSC1*. However, the increased mRNA levels of *KRT1* and *KRT10* after co-treatment with eplerenone were offset by further co-treatment with mifepristone. In addition, we analyzed the effect of mifepristone and eplerenone on keratinocyte differentiation in the basal state (Fig. S2). Mifepristone increased the mRNA levels of *FLG* and *LOR* but decreased those of *KRT1*, *KRT10*, and *DSC1*. In contrast, eplerenone had little effect on the mRNA levels of keratinocyte differentiation. Taken together, mifepristone increased the mRNA levels of *FLG* and *LOR* in both cortisol-treated and basal states; however, eplerenone showed a significant increase in the mRNA levels of *KRT1* and *KRT10* in cortisol-treated NHEKs.

To further elucidate the roles of GR and MR in keratinocyte differentiation, we introduced small interfering RNAs (siRNAs) against GR and MR into the NHEKs and examined the subsequent mRNA levels of keratinocyte differentiation markers (Fig. 3f–j). The reduction in *GR* and *MR* mRNA levels by each siRNA was validated (Fig. 3f, g). However, the siRNAs against GR and MR each also slightly decreased the mRNA levels of the other receptor. Cortisol reduced the mRNA expression of *FLG*, *LOR*, and *KRT1* in non-targeting (NT) siRNA-treated NHEKs (Fig. 3h–j). GR siRNA-treated NHEKs showed higher mRNA levels of keratinocyte differentiation markers than NT siRNA-treated NHEKs in the basal state, whereas those of MR siRNA-treated NHEKs were similar. In GR siRNA-treated NHEKs, cortisol did not decrease the mRNA levels of *FLG*, *LOR*, and *KRT1*, but rather increased them. In contrast, in MR siRNA-treated NHEKs, cortisol decreased the mRNA levels of *FLG*, *LOR*, and *KRT1*. These results indicate that GR in involved in regulating keratinocyte differentiation in the basal status and the cortisol-induced decreases in the mRNA levels of keratinocyte differentiation markers mainly result from the activation of GR rather than MR by cortisol.

### Screening compounds with MR antagonizing properties

To find a compound with MR antagonist properties, which can be utilized as a cosmetic ingredient, we screened 30 single compounds derived from soybeans, green tea, and Korean ginseng. To select a compound that reacts selectively to MR, MR transcriptional activity was measured and compared among the 30 compounds. We first evaluated the expression of MR and GR in MDA-MB-453, CV-1, and MDA KB2 cells (Fig. S3a). Both GR and MR genes were expressed in MDA-MB-453 cells, and MR and GR genes were expressed in CV-1 cells and MDA-KB2 cells, respectively. Therefore, we transfected CV-1 cells, which expressed MR rather than GR, with an MMTV-luciferase reporter plasmid with hexanucleotide 5'-TGTTCT-3' as the enhancer element sequence and examined the effect of the 30 compounds on MR transcriptional activity using a luciferase reporter gene assay (Fig. S3c–h, and Table S1). The MR transcriptional activity values of the compounds were lowest (in ascending order) in epicatechin gallate, epicatechin, L-theanine, and 7,3',4'-trihydroxyisoflavone (7,3',4'-THIF). Considering the cell toxicity and stability of these cosmetic formulations, we selected 7,3′4,'-THIF for further study from among these four compounds with the lowest MR transcriptional activity values. 7,3’,4’-THIF is a hydroxyisoflavone that is daidzein-substituted by a hydroxyl group at position 3’ (Fig. S3b).

**Figure 4:**
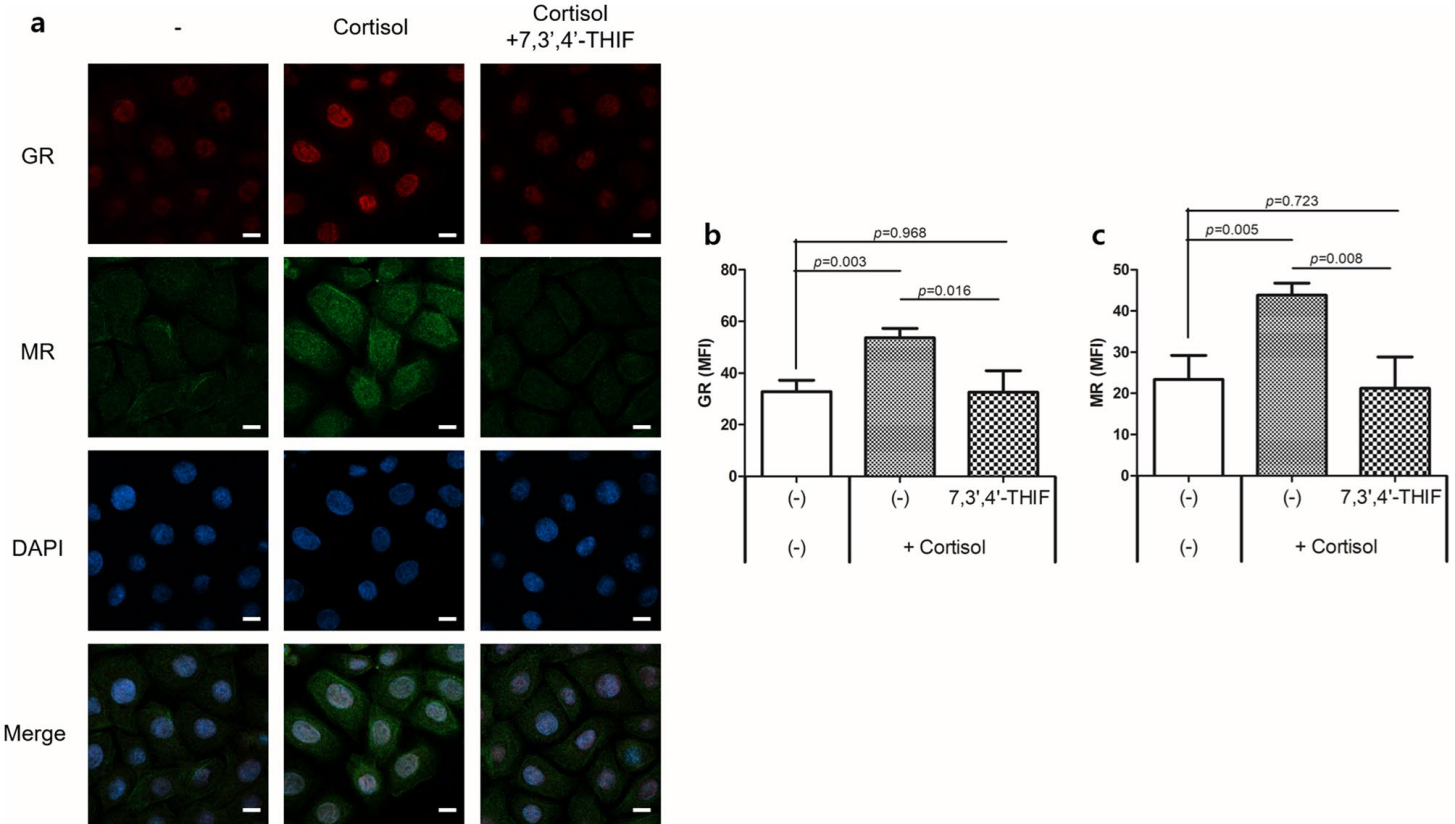
7,3’,4’-THIF inhibited cortisol-induced nuclear translocation of MR and GR in NHEKs. NHEKs were treated with 7,3’,4’-THIF (1 ppm) in the presence of cortisol (10 μM) for 24 h. Cells were stained with antibodies against GR (red) and MR (green) and observed under confocal microscopy. Nuclei were stained with DAPI (blue). (**a**) Representative images. Scale bars, 10 μm. (**b**, **c**) Quantification of mean fluorescence intensity (MFI) of GR and MR in nucleus. Data are expressed as mean ± SD of three independent experiments.

To validate 7,3’,4’-THIF as an MR antagonist in NHEKs, we analyzed the nuclear translocation of MR and GR in NHEKs treated with 7,3’,4’-THIF in the presence or absence of cortisol (Fig. 4 and Fig. S4). In cortisol-treated NHEKs, 7,3',4'-THIF inhibited the nuclear translocation of MR by cortisol. In addition, the nuclear translocation of GR by cortisol was also inhibited by 7,3',4'-THIF, but was less inhibited than that of MR (Fig. 4). In the basal state, 7,3’,4’-THIF treatment did not affect the nuclear translocation of either MR or GR (Fig. S4). These results indicate that, in NHEKs that express both MR and GR, 7,3’,4’-THIF inhibited the nuclear translocation of not only MR but also GR.

### 7,3',4'-THIF inhibits the cortisol-induced decreased expression of keratinocyte differentiation markers

**Figure 5:**
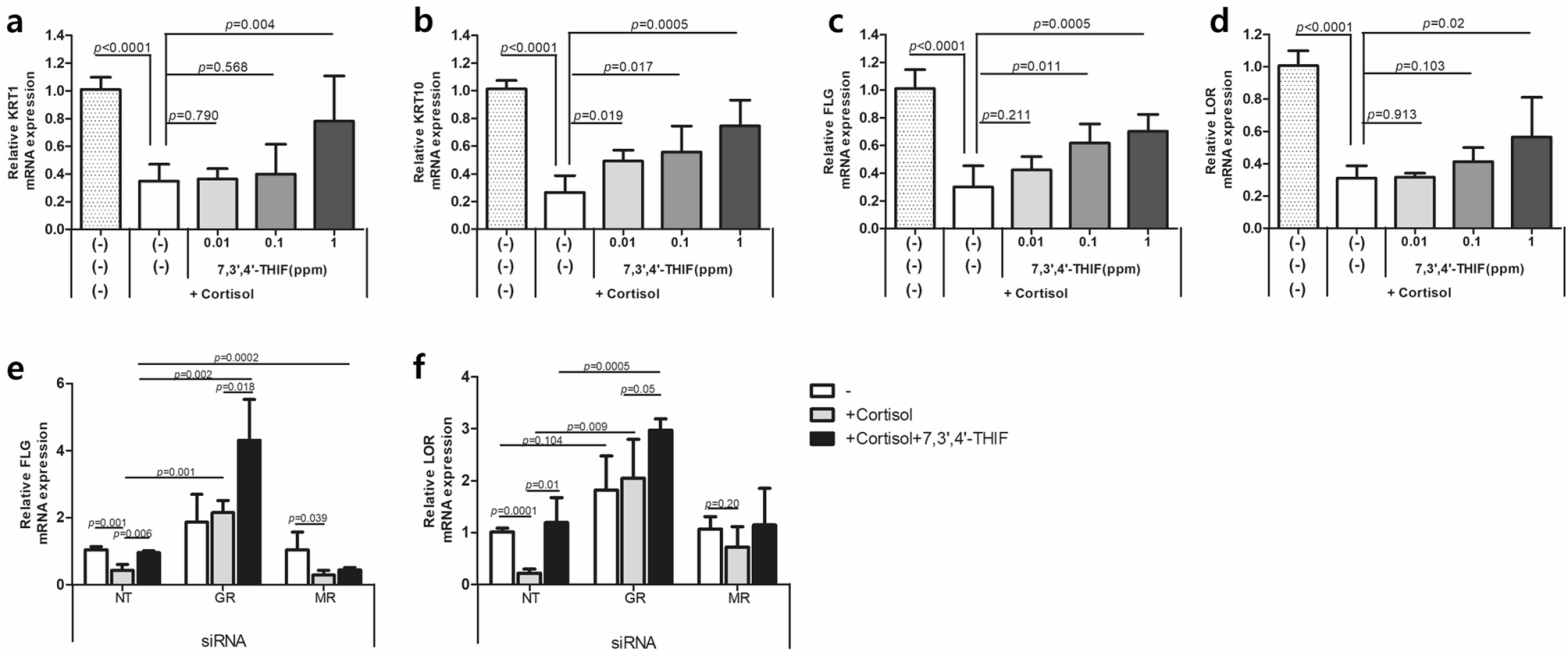
Effect of 7,3’,4’-THIF on mRNA expression of keratinocyte differentiation markers in NHEKs. (**a–d**) NHEKs were treated with 7,3’,4’-THIF (0.01, 0.1, 1 ppm) in the presence of cortisol (10 μM) for 4 days, and the mRNA levels of KRT1, KRT10, FLG, and LOR were analyzed by RT-qPCR. (**e**, **f**) NHEKs were transfected with control (non-targeting; NT), GR, or MR siRNAs. mRNA expression of FLG and LOR in siRNA-expressing NHEKs treated with 7,3’,4’-THIF (1 ppm) in the presence of cortisol (10 μM) for 4 days. mRNA levels were normalized to that of RPL13A. Data are expressed as mean ± SD of three independent experiments.

To determine the effect of 7,3',4'-THIF on skin barrier function, the mRNA levels of keratinocyte differentiation markers such as *KRT1*, *KRT10*, *FLG*, and *LOR* were analyzed after treatment with 7,3',4'-THIF in the presence or absence of cortisol. In cortisol-treated NHEKs, 7,3’,4’-THIF inhibited the cortisol-induced reduction in the mRNA levels of *KRT1*, *KRT10*, *FLG*, and *LOR* in a dose-dependent manner (Fig. 5a–d). In the basal state, 7,3',4'-THIF increased the mRNA levels of *FLG* and *LOR*, but not *KRT1* (Fig. S5).

To elucidate whether 7,3’,4’-THIF inhibits the cortisol effect via GR or MR, we examined the effect of 7,3’,4’-THIF in GR or MR siRNA-treated NHEKs in the presence of cortisol (Fig. 5e and f). In GR siRNA-treated NHEKs, the mRNA expression of *FLG* and *LOR* was further increased by co-treatment with cortisol and 7,3',4'-THIF, compared to when only cortisol was used. In contrast, in MR siRNA-treated NHEKs, there was no significant difference between 7,3’,4’-THIF + cortisol and cortisol only. As GR siRNA-treated NHEKs showed remarkably diminished GR expression (Fig. 3f), cortisol is more likely to activate MR than GR in GR siRNA-treated NHEKs. These results suggest that, in GR siRNA and cortisol-treated NHEKs, co-treatment with 7,3’,4’-THIF increased *FLG* and *LOR* mRNA levels by inhibiting of MR activation. This highlights the role of MR activation by cortisol in cortisol-induced decreases of keratinocyte differentiation, and the role of MR antagonists in preventing it.

**Figure 6:**
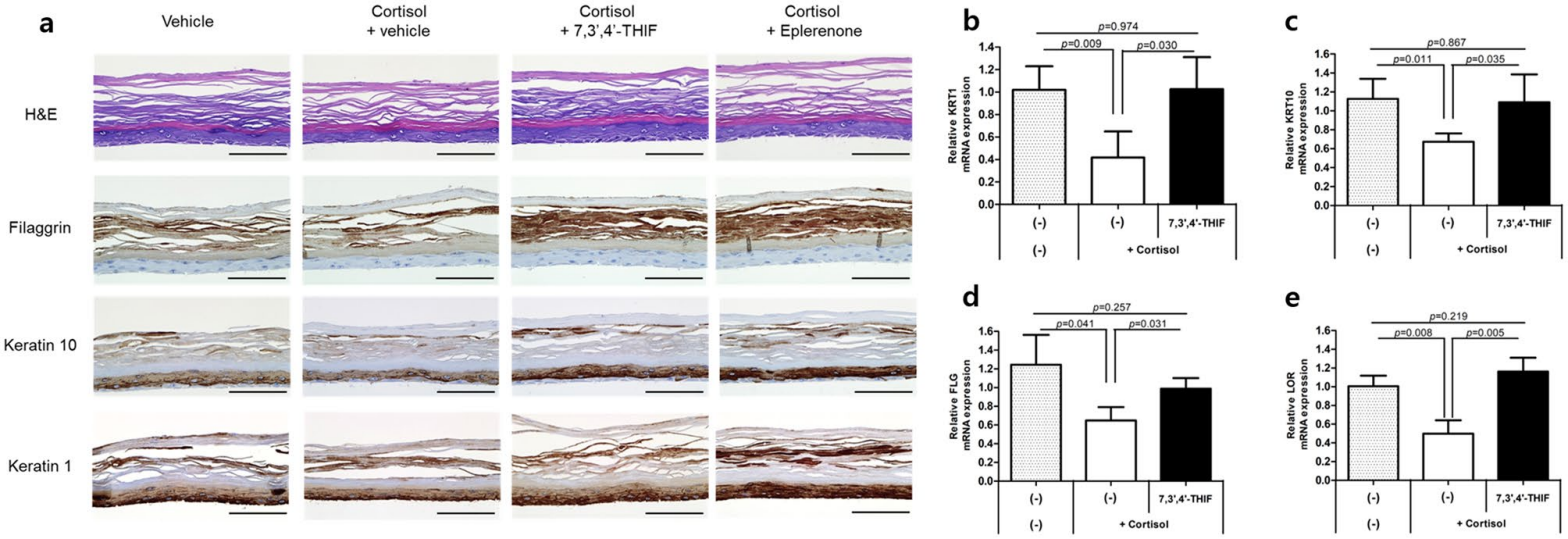
7,3’,4’-THIF ameliorated skin barrier impairment by cortisol in a reconstituted human epidermis (RHE**).** A RHE was topically treated with 7,3′4’-THIF (1 ppm) or eplerenone (MR antagonist, 1 μM) in the presence of cortisol (10 μM) for 6 days. (**a**) Immunohistochemistry analysis using relevant antibodies. H&E, Hematoxylin and eosin staining. Scale bars, 100 μm. Quantitative RT-PCR analysis of (**b**) KRT1, (**c**) KRT10, (**d**) FLG, and (**e**) LOR mRNA levels in the RHE. mRNA levels were normalized to that of RPL13A. Data are expressed as mean ± SD of three independent experiments.

We further examined the effect of 7,3’,4’-THIF on cortisol-induced skin barrier dysfunction in an RHE (Fig. 6). 7,3′4’-THIF (1 ppm), eplerenone (1 μM), or vehicle (0.01% DMSO in phosphate-buffered saline) were applied topically to an RHE in the presence or absence of cortisol (10 μM) every other day for 6 days. Cortisol induced a thinner and less dense epidermal structure (Fig. 6a). The topical application of 7,3’,4’-THIF and eplerenone ameliorated cortisol-induced skin barrier impairment and improved the protein levels of epidermal differentiation markers decreased by cortisol. In the RHE without cortisol treatment, 7,3’,4’-THIF and eplerenone also increased the protein expression of Filaggrin and Keratin 10 (Fig. S6). In addition, the cortisol-induced decrease in the mRNA levels of epidermal differentiation markers was also recovered by 7,3’,4’-THIF (Fig. 6b–e). In summary, 7,3’,4’-THIF treatment ameliorated cortisol-induced decreases in keratinocyte differentiation marker expression and increased their expression in the basal state, in a similar manner to eplerenone.

### Topical application of 7,3',4'-THIF prevents PS-induced skin barrier dysfunction

To verify the effect of 7,3',4'-THIF in improving skin barrier function impaired by PS, clinical experiments were conducted. Twenty-five healthy male medical students were recruited for this study. The mean age of the participants was 19.8 ± 0.9 years (mean ± SD). They applied a 7,3’,4’-THIF (0.1%)-containing cream or vehicle on each side of both forearms for 10 days until the middle of their exam period. Examination stress is an established model of PS that disrupts skin barrier function^9,13,29,30^. The experiments were approved by the Institutional Review Board of Wonju Severance Christian Hospital and were carried out in accordance with their guidelines and regulations. Informed consent was obtained from all participants.

**Figure 7:**
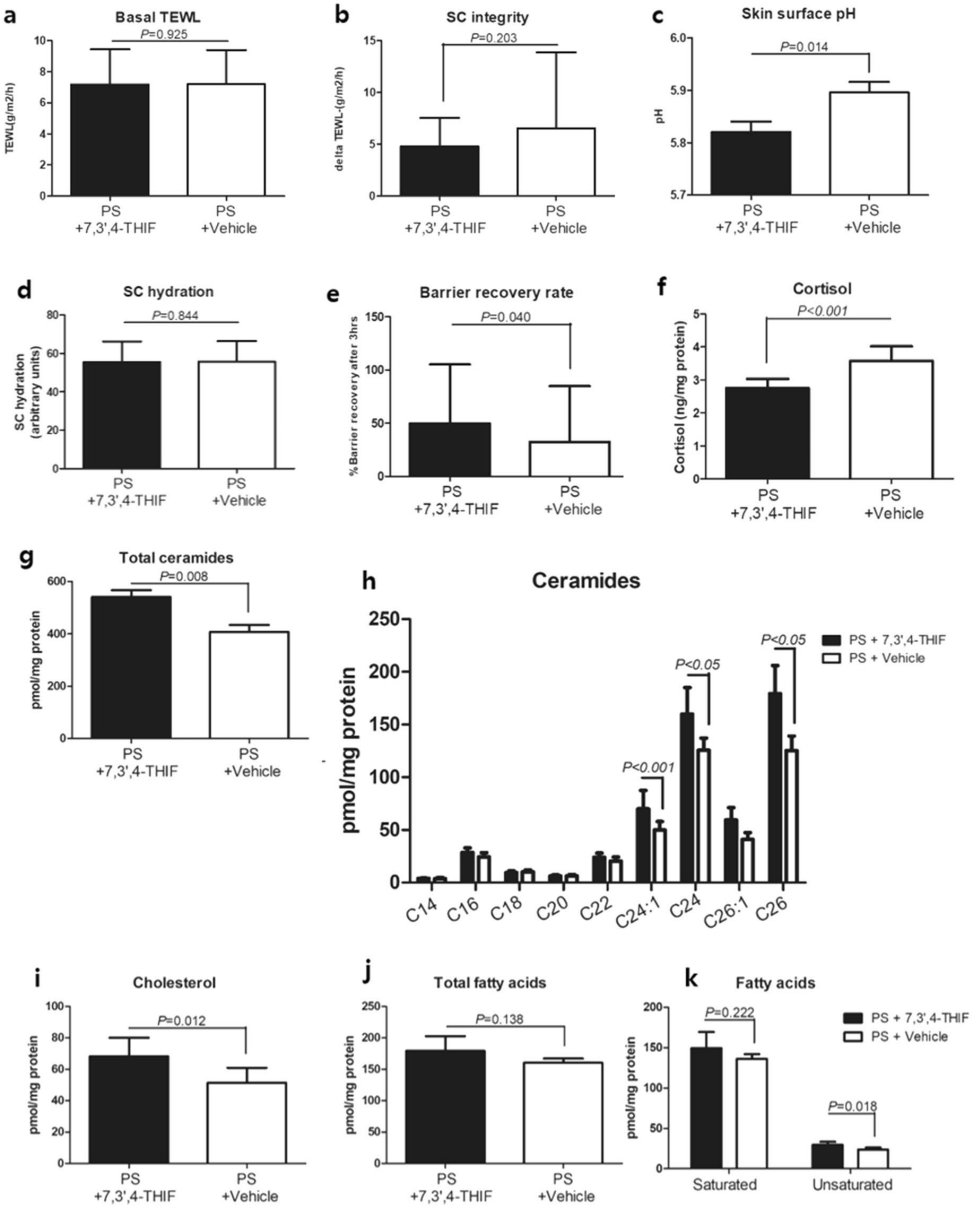
Topical application of 7,3',4'-trihydroxyisoflavone (7,3’,4’-THIF), a novel mineralocorticoid receptor antagonist, improves psychological stress (PS)-induced barrier impairment and increases the amounts of stratum corneum (SC) lipids. Comparison of basal transepidermal water loss (TEWL) (**a**), SC integrity (**b**), skin surface pH (**c**), SC hydration (**d**), and barrier recovery rate (**e**) between 7,3’,4’-THIF-treated sides and vehicle-treated sides in 25 medical students under PS. Quantitative analysis of cortisol (f), total ceramides (g), ceramides with specific chain length (**h**), cholesterol (**i**), total fatty acids (**j**), and saturated and unsaturated fatty acids (**k**) using tape-stripped skin samples from the participants. Data are expressed as mean ± SD (a-f; N = 25, g-k; N = 6). Paired t-test and Wilcoxon signed-rank test were used as appropriate.

After 10 days of topical application, in the middle of the examination period, the skin barrier function of each forearm was measured and compared. Topical application of the 7,3’,4’-THIF-containing cream significantly lowered skin surface pH (*p* = 0.014) and improved barrier recovery (*p* = 0.040) compared to the vehicle cream (Fig. 7a–e). Skin surface pH plays a crucial role in maintaining skin barrier function, and a lowered skin surface pH indicates improved of skin barrier function^31^. Basal TEWL, SC integrity, and SC hydration did not differ between the two sides. The SC cortisol levels in tape-stripped samples were significantly lower in the 7,3’,4’-THIF group than in the vehicle group (*p* < 0.001) (Fig. 7f).

To investigate the effect of 7,3’,4’-THIF on permeability barrier homeostasis, the amounts of SC lipids were analyzed. Using tape-stripped SC samples from both sides, three main SC lipids (ceramides, cholesterol, and fatty acids) were quantified. Topical application of 7,3’,4’-THIF significantly increased the amount of ceramides (*p* = 0.008) and cholesterol (*p* = 0.012). In particular, very long-chain ceramides, such as C24:1, C24:0, C26:1, and C26:0, were preferentially increased. Unsaturated FAs were increased (*p* < 0.05), whereas total FAs were not (Fig. 7g–k). In summary, topical application of 7,3’,4’-THIF in PS participants improved skin barrier functions, such as skin surface pH and barrier recovery rate, which were attributed to increased SC lipids.

## Discussion

Robust evidence supports that exogenous GC and endogenous GC under PS negatively affect the skin barrier function. Among various psychological stressors, examination stress is a validated model of PS, as shown in previous studies^9,13,29,30^. PS upregulates the HPA axis to stimulate systemic stress hormone production^12^ and 11β-HSD1 activity in keratinocytes^14^. An elevated concentration of cortisol in the skin is a key mediator of PS-induced skin barrier dysfunction. It causes decreased production of epidermal lipids and structural proteins, increased skin surface pH, and decreased corneodesmosome density, eventually deteriorating the skin barrier function characterized by decreased SC integrity and delayed barrier recovery^1,10,13^. In this study, we aimed to demonstrate the action of cortisol inside keratinocytes, which is hypothesized to be mediated by GR and MR. Furthermore, we investigated whether MR antagonists can ameliorate GC-induced skin barrier dysfunction in NHEKs, an RHE, and clinical studies.

Previous studies regarding MR antagonism against GC excess have demonstrated that it prevents delayed wound closure and epidermal atrophy by enhancing keratinocyte proliferation, migration, and differentiation^25,26^. Our results can be understood in the same context. Skin barrier function is organized by epidermal proliferation and differentiation, which begins at the basal layer^32,33^. In this study, we suggest further evidence of inappropriate GC-induced MR activation by illustrating that GC increases the nuclear translocation of MR, as well as GR. GR and MR are ligand-inducible transcription factors^19^. After steroid hormones are bound, these receptors translocate from the cytoplasm into the nucleus and exhibit their transactivation potential.

We observed that mifepristone and eplerenone decreased the nuclear translocation of their respective receptors in cortisol-treated NHEKs (Fig. 2). In addition, co-treatment of mifepristone or eplerenone with cortisol offset the cortisol-induced reduction in the mRNA levels of keratinocyte differentiation markers (Fig. 3a–e). However, in the basal state, single treatment with mifepristone increased the mRNA levels of *FLG* and *LOR* (Fig. S2). Such remarkable changes were not observed with eplerenone. In addition, in siRNA studies, treatment with GR siRNA alone enhanced the mRNA expression of *FLG*, *LOR*, and *KRT1*, which was not observed with MR siRNA (Fig. 3h–j). In GR siRNA-treated NHEKs, cortisol treatment did not decrease the mRNA levels of *FLG*, *LOR,* and *KRT1*. Furthermore, in GR siRNA- and cortisol-treated NHEKs, 7,3’,4’-THIF further increased the mRNA levels of *FLG* and *LOR*, which was not observed in MR siRNA-treated NHEKs (Fig. 5e and f). This could be attributed to 7,3’,4’-THIF inhibiting the MR activation by cortisol in the absence of GR. Taken together, GR is involved in regulating keratinocyte differentiation in both basal and cortisol-excess states. In contrast, MR is activated when excess cortisol is administered, and is involved in the cortisol-induced suppression of keratinocyte differentiation.

7,3’,4’-THIF is a compound with the property of an MR antagonist, selected after screening 30 compounds, according to their inhibition of MR transcriptional activity (Table S1 and Fig. S3). It is a metabolite of daidzein, a representative isoflavone found in soybeans. A recent study suggested that 7,3’,4’-THIF suppresses α-melanocyte-stimulating hormone-induced melanogenesis by targeting melanocortin 1 receptor in B16F10 cells^34^.

The inhibition of epidermal lipid synthesis is one of the key deleterious effects of GC, which deteriorates permeability barrier homeostasis. Topical application of physiologic SC lipids, comprising equimolar ceramides, cholesterol, and free FAs, prevents barrier disruption in both PS and topical GC-treated mice^1,15,35^. In this study, SC lipids were analyzed in two clinical studies. Topical application of spironolactone, an MR antagonist, increased the amounts of ceramides and cholesterol in clobetasol propionate-treated skin (Fig. 1f–h). Topical application of a 7,3’,4’-THIF-containing cream increased the amounts of ceramides and cholesterol in PS participants (Fig. 7g–k). In particular, the levels of long chain ceramides (C24 to C26), which are essential for skin barrier function^36–38^, were increased, while those of ceramides with shorter carbon chains were not significantly changed (Fig. 7h).

Skin barrier function was improved by topical application of spironolactone (Fig. 1a–e) and 7,3’,4’-THIF-containing cream (Fig. 7a–e), which was attributed to increased keratinocyte differentiation and SC lipids^32,39^. Although topical spironolactone only improved SC integrity and the 7,3’,4’-THIF-containing cream improved only the pH of the skin surface and barrier recovery rate, not all the measured parameters, these changes are important for the improvement of skin barrier function. An acidic skin surface pH plays a crucial role in maintaining permeability barrier homeostasis^31^. SC integrity and barrier recovery rate are challenge tests for skin barrier function, measuring the extent to which the permeability barrier is damaged after 15 consecutive tape-stripping cycles and how much it recovers after 2 h^40^. However, basal TEWL and SC hydration were measured when the skin barrier was not challenged. The participants had no skin diseases. Increased basal TEWL and decreased SC hydration are common findings in patients with impaired skin barriers or severe dry skin, which is one of the exclusion criteria of our clinical trials.

Another method of regulating MR activation by GC is through the pre-receptor regulating the activity of 11β-HSD1^19^. Under various stimuli, including ultraviolet irradiation, aged skin, and exogenous GC and PS, the activation of 11β-HSD1 increases the local production of the active GC cortisol, thus stimulating MR downstream^13,41,42^. The inhibitory intervention of 11β-HSD1 or MR yielded similar results in augmenting wound healing and preventing skin atrophy, aging, or skin barrier dysfunction^43,44^. Therefore, the simultaneous inhibition of both effectors may provide more effective prevention of GC-mediated cutaneous adverse effects under various stimuli. However, the inhibition of 11β-HSD1 may also have negative effects, depending on the condition of the skin. In an oxazolone-induced mouse model of AD, inhibition of 11b-HSD1 aggravated the development of AD and increased the serum cytokine levels associated with AD^45^. Therefore, the potential negative effects of 11β-HSD1 inhibition may induce in AD. In this study, we tried to improve skin response by suppressing the stress response using MR antagonists in healthy people, not in those with inflammatory skin diseases such as AD. We also confirmed that 7,3’,4’-THIF did not convert cortisone to cortisol in NHEKs (Fig. S7).

Further investigation is required to address the alteration in the anti-inflammatory effects of GC by MR antagonists. When canrenoate, an MR antagonist, is co-applied with clobetasol in an irritated human skin equivalent model, among the anti-inflammatory properties of GC, the inhibition of COX-2 and upregulation of the anti-inflammatory cytokine interleukin (IL)-10 are preserved, whereas the inhibition of pro-inflammatory cytokines, such as IL-6 and IL-1α, is not^46^. Applying MR antagonists to inflamed skin (e.g., AD) could raise concerns about modulating the anti-inflammatory property of GCs or perhaps aggravating skin inflammation. However, in this study, the skin conditions we aimed to treat were those of topical GC-treated or PS subjects who had weakly increased cortisol and no apparent signs of inflammation.

For patients with cutaneous dermatoses where PS acts as an aggravating factor, an investigation into whether the MR blockade can prevent aggravation by PS is needed. We observed that the keratinocyte differentiation markers increased by co-treatment with mifepristone were *FLG*, *LOR*, and *DSC1*, but not *KRT1* and *KRT10*, which were improved by co-treatment with eplerenone. *FLG* and *LOR* are late differentiation markers, and *KRT1* and *KRT10* are early differentiation markers^47^. Therefore, further investigation into the involvement of GR and MR in late and early keratinocyte differentiation is needed.

A limitation of our study is that the participants were all healthy male volunteers. In the preliminary clinical trial, spironolactone was used as an MR antagonist because topical spironolactone has been commercialized as a product, so called ‘S5 cream’, for the treatment of pattern hair loss and acne. However, eplerenone was used in the NHEKs and RHE studies because of its higher MR selectivity than spironolactone^26,48^. This difference in the chemicals used in the clinical and laboratory studies resents another limitation. In addition, to demonstrate the efficacy of 7,3’,4’-THIF in improving skin barrier function in clinical studies, it was compared to a control vehicle, not to a pre-existing MR antagonist, such as topical spironolactone.

In conclusion, under PS or topical GC treatment, excess GC activates MR as well as GR, resulting in skin barrier dysfunction. Topical application of MR antagonists significantly prevented the deleterious effects of PS or exogenous GC on the skin barrier by improving keratinocyte differentiation and SC lipid synthesis. This could represent a promising therapeutic option to ameliorate skin disorders caused by PS or skin fragility caused by the prolonged use of systemic or topical GCs.

## Materials and methods

### Human studies

The experiments were approved by the Institutional Review Board of Wonju Severance Christian Hospital (CR319140 and CR317125) and were carried out in accordance with the appropriate guidelines and regulations. Informed consent was obtained from all subjects.

#### Application of topical GC and spironolactone in healthy participants

A total of 11 healthy young adults were recruited. The same sites on both forearms of each participant were treated with 0.05% clobetasol propionate ointment (Dermovate, GlaxoSmithKline, Uxbridge, UK), 5% spironolactone cream (Shanghai Sunshine Technology, Shanghai, China), or 0.05% clobetasol propionate ointment + Cetaphil lotion (Galderma Laboratories, Fort Worth, TX, USA) for five consecutive days. Skin barrier functions and the amounts of SC lipids in the tape-stripped samples were measured on each forearm.

#### Application of a novel MR inhibitor, 7,3',4'-THIF, in humans under PS

A total of 25 medical students were recruited for this study. All participants were male medical students of the same grade and were all in good health. An examination model was adopted as the type of PS. The same sites on both forearms of each participant were treated with 7,3’,4’-THIF (0.1%)-containing cream or vehicle moisturizer for 10 consecutive days before the examination period. During the examination period, skin barrier functions and the amount of SC lipids in the tape-stripped samples were measured on each forearm.

### Cell culture

NHEKs (Lonza, Basel, Switzerland) within two or three passages were cultured in KBM-Gold medium supplemented with a KGM-Gold Bullet Kit (Lonza) at 37 °C and 5% CO_2_. Hydrocortisone, one of the components of the KGM-Gold Bullet kit, was not added to the medium for the assay. NHEKs were treated with cortisol (10 μM) in the presence or absence of 7,3’,4’-THIF (Indofine Chemical Co., Inc., Hillsborough, NJ, USA), mifepristone (Tocris, Bristol, UK), or eplerenone (Tocris), and harvested 4 days later for further analysis.

### Immunocytochemistry and immunohistochemistry

NHEKs were treated with cortisol with or without mifepristone, eplerenone, or 7,3’,4’-THIF, stained with anti-GR antibody (BD Bioscience, San Jose, CA, USA) or anti-MR antibody (Abcam, Cambridge, MA, USA), and analyzed under a confocal microscope (LSM 700; Carl Zeiss, Jena, Germany). The images obtained were quantified for mean fluorescence intensity (MFI) using ImageJ software (https://imagej.nih.gov/ij). For immunohistochemical analysis, replicate sections from an RHE were stained with the following antibodies: anti-keratin 10 (Biolegend, San Diego, CA), anti-keratin 1 (Biolegend), and anti-filaggrin (Abcam). The detailed protocols for the immunological analyses are described in the Supplementary Information.

### Quantitative real-time PCR

Total RNA was prepared using TRIzol (Thermo Fisher Scientific, Waltham, MA, USA), and cDNA was synthesized using a Revertaid RT kit (Thermo Fisher Scientific). Quantitative real-time PCR was performed using TaqMan probes (described in the Supplementary Information; Thermo Fisher Scientific) and the 7500 Fast Real-Time PCR system (Thermo Fisher Scientific). The RPL13A gene was used to normalize the amount of cDNA. Relative differences in gene expression were calculated from the threshold cycle (Ct) values. At least three independent experiments were assessed to calculate mean values ± SD.

### siRNA experiments

NHEKs were transfected with 50 nM of non-targeting control siRNA or siRNAs against GR or MR (Dharmacon, Lafayette, CO, USA), using Lipofectamine RNAimax transfection reagent (Invitrogen, Carlsbad, CA, USA), according to the manufacturer’s instructions.

### MR transactivation assay

CV-1 cells were seeded into 24-well plates and cultured for 24 h before transfection. Prior to transfection, the medium was replaced with 10% charcoal dextran-treated FBS–DMEM. After 4 h, a DNA mixture containing an MMTV-luciferase reporter plasmid (pGL4.36[*luc2P*/MMTV/Hygro] vector, 0.3 μg, Promega) with (TGTTCT)_6_ as the enhancer element sequence, and an internal control plasmid pRL-SV-40 (5 ng), was transfected using the TransFast reagent (Promega, Madison, WI, USA). After transfection, cells were treated with 10 μg/mL compounds (samples) or 1 μM spironolactone. After 2 h, the treated cells were further treated with 1 nM dexamethasone and incubated for an additional 24 h. The luciferase activities of the cell lysates were measured using the Dual-Luciferase Reporter Assay System, according to the manufacturer’s instructions (Promega). The relative luciferase activity was normalized to the corresponding Renilla luciferase activity to determine transfection efficiency.

### Reconstructed human epidermis

A RHE was purchased from MatTek (Ashland, MA, USA) and maintained according to the manufacturer’s instructions. The RHE was systemically treated with cortisol (10 μM), and topically treated with 7,3’,4’-THIF (1 ppm), eplerenone (1 μM), or vehicle (0.01% DMSO in phosphate-buffered saline) every other day for 6 days.

## Supplementary Information

Supplementary Information.

## Abbreviations

PS: Psychological stress
7,3’,4’-THIF: 7,3',4'-Trihydroxyisoflavone
TEWL: Transepidermal water loss
SC: Stratum corneum
GR: Glucocorticoid receptor
MR: Mineralocorticoid receptor
GC: Glucocorticoid
FA: Fatty acid
11β-HSD1: 11β-Hydroxysteroid dehydrogenase type I
11β-HSD2: 11β-Hydroxysteroid dehydrogenase type II
